# Patient and Provider Attitudes Toward the Use of Patient Portals for the Management of Chronic Disease: A Systematic Review

**DOI:** 10.2196/jmir.3703

**Published:** 2015-02-20

**Authors:** Clemens Scott Kruse, Darcy A Argueta, Lynsey Lopez, Anju Nair

**Affiliations:** ^1^School of Health AdministrationCollege of Health ProfessionsTexas State UniversitySan Marcos, TXUnited States

**Keywords:** electronic health record (EHR), health information technology (HIT), internet, patient portal, chronic disease, disease management, self-management

## Abstract

**Background:**

Patient portals provide patients with the tools to better manage and understand their health status. However, widespread adoption of patient portals faces resistance from patients and providers for a number of reasons, and there is limited evidence evaluating the characteristics of patient portals that received positive remarks from patients and providers.

**Objective:**

The objectives of this systematic review are to identify the shared characteristics of portals that receive favorable responses from patients and providers and to identify the elements that patients and providers believe need improvement.

**Methods:**

The authors conducted a systematic search of the CINAHL and PubMed databases to gather data about the use of patient portals in the management of chronic disease. Two reviewers analyzed the articles collected in the search process in order remove irrelevant articles. The authors selected 27 articles to use in the literature review.

**Results:**

Results of this systematic review conclude that patient portals show significant improvements in patient self-management of chronic disease and improve the quality of care provided by providers. The most prevalent positive attribute was patient-provider communication, which appeared in 10 of 27 articles (37%). This was noted by both patients and providers. The most prevalent negative perceptions are security (concerns) and user-friendliness, both of which occurred in 11 of 27 articles (41%). The user-friendliness quality was a concern for patients and providers who are not familiar with advanced technology and therefore find it difficult to navigate the patient portal. The high cost of installation and maintenance of a portal system, not surprisingly, deters some providers from implementing such technology into their practice, but this was only mentioned in 3 of the 27 articles (11%). It is possible that the incentives for meaningful use assuage the barrier of cost.

**Conclusions:**

This systematic review revealed mixed attitudes from patients and their providers regarding the use of patient portals to manage their chronic disease. The authors suggest that a standard patient portal design providing patients with the resources to understand and manage their chronic conditions will promote the adoption of patient portals in health care organizations.

## Introduction

As of 2012, about half of all adults in the United States suffer from one or more chronic diseases [1]. The top 10 chronic conditions are hypertension, coronary heart disease, stroke, diabetes, cancer, arthritis, and hepatitis [1,2]. Chronic conditions affect any individual regardless of age, race, or socioeconomic status, although it was noted that co-morbidity increases with age and prevalence is higher among non-Hispanic white adults [1]. Individuals suffering from more than one chronic disease usually have multiple providers and consume more medical services such as hospitalizations, office visits, and medications, which lead to higher health expenditures [2,3].

The concept of a patient portal has asserted its presence in literature for the last several years. The US government provides a rather clear definition of a patient portal: “a secure online website that gives patients convenient 24-hour access to personal health information from anywhere with an Internet connection” [4]. The patient portal differs from a personal health record (PHR), however, in terms of ownership. The data in a patient portal are owned and managed by the health care organization along with the electronic health record (EHR) [4]. The advantage of a portal over a PHR is that the data are updated whenever there are updates on the EHR, while the data in a PHR are only updated when the patient updates them. Patient portals offer many features, and health care organizations can choose different features of even the same vendor solution. The basic portal enables a patient to access his/her information such as recent office visits, discharge summaries, medications, immunizations, allergies, and lab results, and the more advanced portals enable a patient to request prescription refills, schedule non-urgent appointments, and exchange secure messaging with his/her provider [4].

Features enabled by patient portals are intended to improve quality and access to health care by engaging patients to be more active in managing and monitoring their health [3-6]. Many health care systems have piloted or implemented patient portals with emphasis on secure communication to assist patients with the management of their own health and to improve the coordination of care across multiple providers [3,7,8]. Patients may communicate electronically with their provider, access personal health records (PHR), receive lab results, request for medication refills, schedule appointments, and learn more about their health [7,9,10]. Some portals allow patients to monitor their own health by entering their daily blood sugar levels or weight loss progress, which give patients a greater sense of empowerment in the management of their conditions [10-12].

In 2009, less than 5% of hospitals utilized a Web-based patient portal [13]. Since Congress passed the Health Information Technology for Economic and Clinical Health Act (HITECH) in 2009, patient portal adoption has gained greater attention as it enables a secure means of continuous patient-centered care [3,8,10,11]. Innovations in health information technologies (HIT) allow providers to implement electronic PHRs to deliver targeted patient education for disease management and to support provider decision-making [14-16]. Patient health coaching has emerged as an effective service to educate patients on their chronic conditions, provide eVisits, and strengthen the patient and provider relationship [17-19].

Current research of patient portals has revealed mixed feelings among patients and providers who use Web-based patient portals to monitor their chronic conditions [20]. Despite potential advantages to providing personalized patient-centered care, health care providers are concerned about the increasing workloads to meet patient demands, lost profits, insufficient security, and the high cost of acquiring and maintaining a patient portal system [8,13,16,21].

The purpose of this research is to conduct a systematic literature review to identify provider/patient attitudes toward the use of patient portals for the management of chronic disease. The review will also identify portal features that received favorable responses from patients and providers, and it identifies the portal services that patients and providers find valuable but believe need improvement.

## Methods

### Overview

**Figure 1:**
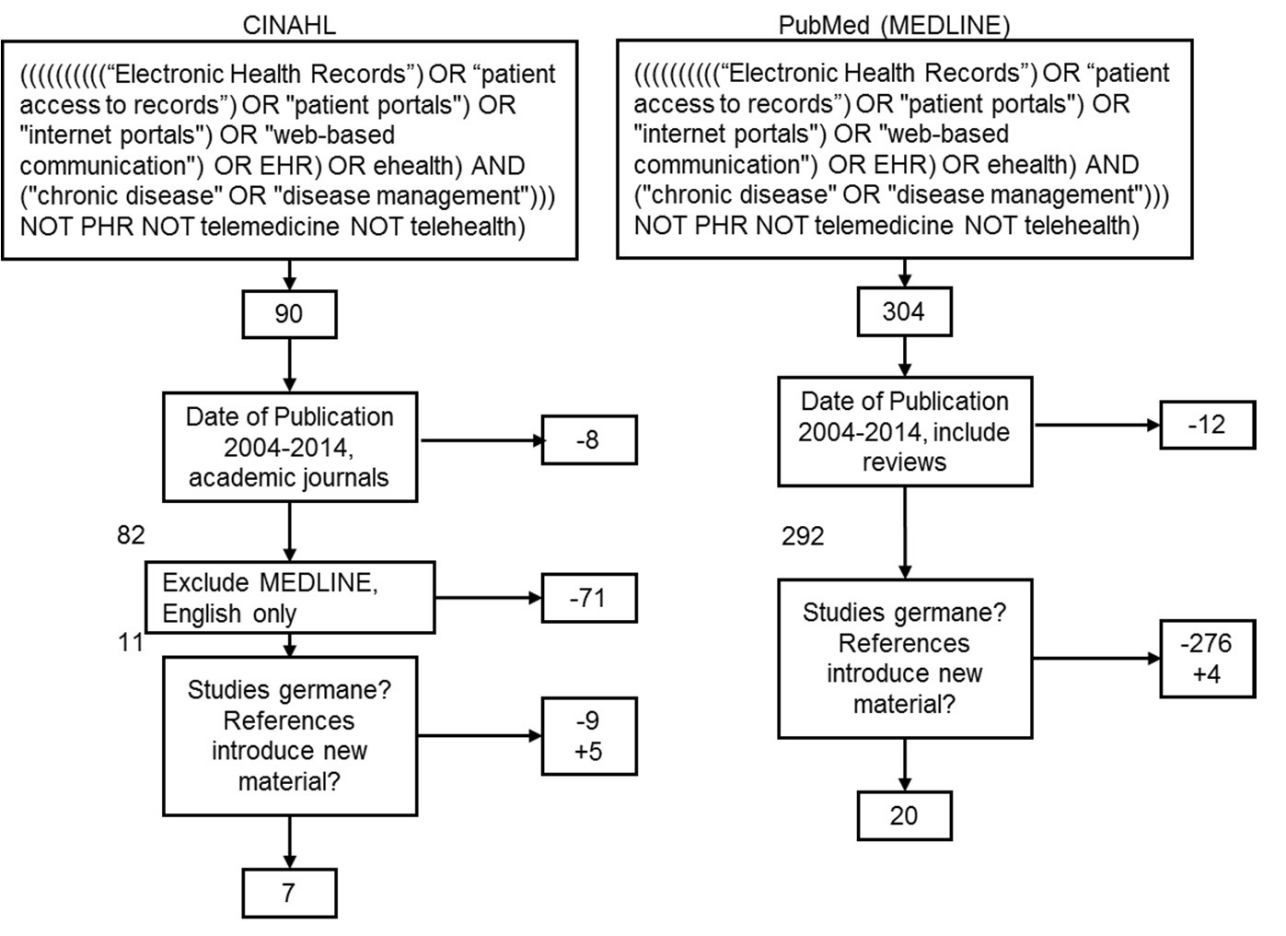
Literature review process.

The search and selection process of the articles used for this review are illustrated in Figure 1. The authors conducted a systematic search using PubMed and CINAHL research databases. A conscious decision was mutually made between researchers to omit Google Scholar in the search because it has an extremely primitive filter capability. The number of key search terms, even when incorporating Boolean operators, creates a highly complex query. Qualitative and quantitative studies and reviews published between January 2004 and July 2014 were included to increase the chance of capture of academic articles on the topic. The broad search terms used included “patient portals”, “internet portals”, “web-based communication”, and “chronic disease”. These terms were chosen from MeSH. Quotation marks for exact phrases and Boolean search operators were included. Because PubMed queries MEDLINE, we excluded MEDLINE from the CINAHL search. The initial search yielded 394 results.

### Exclusion Criteria

Filters were applied to exclude articles outside the period of study (2004-2014) and those not published in academic, peer-reviewed journals. Because PubMed automatically queries MEDLINE, a filter was used in CINAHL to exclude MEDLINE results. A final filter on CINAHL excluded all studies except those published in English. These filters removed 91 articles.

The remaining 303 articles were examined by at least one reviewer. A determination was made whether the article was germane to the study. This exclusion process was entirely manual, and it removed 285 articles. Of the articles excluded from the study, some only included patient portals as a small part of a broader topic of technological advances in patient care, while others focused on EHRs in conjunction with patient portals. One of the articles excluded from the literature review was a comparative study of various portal systems, which did not comment on the patient or provider attitudes toward the use and adoption of a portal system. Our screening criteria primarily revolved around our research question concerning the attitudes of patients and providers toward the use of patient portals for the management of chronic disease. Articles not related to the objective of this literature review were excluded. The remaining articles and their references were examined by at least two reviewers. An additional nine articles were added to the study from the references, but only if they fell within the 10-year date range. The final sample was 27. A table was built to summarize the observations from the authors on the 27 articles under study.

## Results

The wide search criteria enabled a well-rounded evaluation of patient portals across multiple chronic diseases: diabetes, obesity, heart health, cancer, etc. Not surprisingly, there are both positive and negative attitudes presented by patients and providers using a patient portal or a Web-based communication system.

A total of 27 articles were carefully read for common themes. At least two reviewers made and compared notes on the articles for consensus. A more detailed summary of the individual articles is provided in Table 1.

An affinity matrix has been used by other research to illustrate frequency of mention or discussion of a particular topic [22]. For this review, an affinity matrix was created to identify the occurrences of both positive and negative aspects in the literature. This matrix can be found in Table 2. Overall, seven positive qualities and eight negative qualities of patient portals were common threads throughout the literature. In all, there were approximately 105 instances of both positive and negative perceptions of the patient portals.

A total of 11 out of 27 articles (41%) reported an improvement of patient-provider communication as a result of using a patient portal [4,5,7,15,16,18,19,23-26]. Ten of 27 articles (37%) reported a positive association with the secure messaging offered by the patient portal [4,5,7,12,18-20,23-25]. Ten out of 27 articles (37%) mentioned improvements in quality of care as reported by both patient and provider [4,5,7,13,16,18,20,23,25,27]. Ten of 27 articles (37%) reported an increase in disease outcomes as a result of using the patient portal. Nine out of 21 articles (33%) attributed greater self-management of chronic conditions through the presence of educational resources presented through a patient portal [4,7,12,13,16,18,21,23,28]. Seven out of 27 articles (26%) reported from both patients and providers of the ease of navigation and user-friendliness of the portals [4,13,15,21,23,26,28].

Several positive and negative attributes overlapped within the same study. For instance, while the respondents perceived an element from their patient portal as being beneficial, other respondents had a negative experience with a similar element in their portals. Even though patients and providers view secure messaging capabilities in patient portals as a beneficial attribute, 11 of the 27 articles (41%) stated that there was insufficient security in the portal design [7,8,10,12,15,16,20,24,25,27,29]. Also in 11 of 27 articles, patients did not perceive the patient portal as user-friendly and had difficulty navigating Web applications due to a lack of patient technical support, education, and access to the Internet [6,8-10,16-18,21,28-30].

Secure messaging and time management were both mentioned in five of the 27 articles (19%). The latter was most often mentioned by providers as an expression of frustration that they would not have sufficient time to take care of business that is reimbursable. Surprisingly, only three in 27 articles (11%) identified cost as a concern [7,8,14]. This is a surprise because cost is mentioned consistently in the literature relating to cost of other aspects of health information technology [31]. Three of 27 articles (19%) reported a sharp decrease in patient to provider communication after implementing a patient portal due to patients cancelling office visits [10,19,27]. Although patients value the educational resources provided in their patient portal, in three articles, many patients reported difficulty understanding and navigating interactive resources such as health libraries in their patient portal [9,10,15]. Only two of 27 articles (7%) reported negative medical outcomes as a result of using a patient portal [4,5].

**Table 1:** Summarized findings of the literature.^a^

Title	Findings
Primary-care physician attitudes towards the use of a secure web-based portal designed to facilitate electronic communication with patients [32].	Prior to using the patient portal, physicians demonstrated concern of work overload, lower reimbursement, and issues of security. After using the patient portal, physicians reported time savings, ease of documentation, improved quality of patient care, and improved communication.
Enhancing access to patient education information: a pilot usability study [33].	Patients learned more about their disease and how to manage it with the help of the educational links in their patient portal.
DIADEM: Implementation of a comprehensive disease management programme for type 2 diabetes [30].	User response was very positive. Patients entered their own glucose information into the Web-based interface
Interest in the use of computerized patient portals: role of the provider-patient relationship [13]	There was dissatisfaction in the provider-patient relationship with the use of the patient portal. Providers were not satisfied with its communication capabilities or responsiveness, and they reported having difficulty obtaining patient specific medical information.
Measuring the impact of patient portals: what the literature tells us [9]	The implementation of patient portals decreased office visits and increased the number of telephone calls and email from patients.
Patient use of secure electronic messaging within a shared medical record: A cross sectional study [10]	Patients over the age of 65 and covered by Medicaid are less likely to use secure messaging due to problems understanding the information, difficulty using technology, physical disabilities, and inability to access the Internet.
The new age of healthcare communications [16]	Portals increase the use of email communication, online appointment scheduling, and electronic health records among patients. Physicians are concerned about the loss of profitability that results from heavy use of portals, the breach of patient privacy, and the increased workload in responding to patient emails.
Health coaching via an internet portal for primary care patients with chronic conditions: a randomized controlled trial [20]	Patients experienced a higher quality, more informative clinic visit after using a patient portal because they were better informed about their health.
Usability testing finds problems for novice users of pediatric portals [28]	Despite prior heuristic testing, users found navigation of a portal to be difficult; however, it is clear that portals have the potential to assist in making health care system interfaces for laypersons more user-friendly and functional.
The literacy divide: health literacy and the use of an internet-based patient portal in an integrated health system-results from the diabetes study of northern California (DISTANCE) [8]	There is a distinction among users and non-users with respect to health literacy, educational resources, and ability to navigate and use the technology effectively.
Patient web portals to improve diabetes outcomes (systematic review) [18]	A review of 26 articles illustrates the value of patient portals to both patient and provider. Portals have a positive effect on outcomes of users.
Factors influencing the use of a web-based application for supporting the self-care of patients with type 2 diabetes: a longitudinal study [19]	Web-based applications improve patient access to care and enhanced the patient-nurse communication process. Timely feedback from providers allowed patients to better manage their diabetes.
Patient reported barriers to enrolling in a patient portal [29]	User training must include the value of different features of a portal, and reminders should be sent often.
Variation in use of internet-based patient portals by parents of children with chronic disease [26]	Only 15.9% of portal users were still using the portals after 3 months of initial registration. Education about the benefits of the portal is necessary for patients to fully understand the value of portals in patient care.
Impact of health portal enrollment with email reminders on adherence to clinic appointments: a pilot study [34]	Portal users were more engaged with their own care. When the healthcare organization combined email reminders with the portal use, monthly no-show rates were significantly reduced across multiple clinics.
Improving diabetes management with a patient portal: a qualitative study of diabetes self-management portal [12]	Patients were satisfied overall with features presented in the portal: users stated that they were more aware of their health status. The study stated that some portal features were too difficult for the patients to understand and navigate.
Internet use by primary care patients: Where is the digital divide [24]	Internet use is high among the sample (n=777). Major difference between users with chronic conditions was age. Older generations need more training.
The impact of electronic patient portals on patient care: a systematic review of controlled trials [31]	There are very few scientific studies that examine the relationship of portal use to health outcomes or patient empowerment. There is insufficient evidence to suggest any relationship, positive or negative.
A national action plan to support consumer engagement via ehealth [5].	The use of eHealth can augment patient engagement, improve individual health, and achieve broader health care system improvements. Patient users of patient portals feel better prepared for the medical encounter, as relevant questions, are better informed about their health, and are more likely to take steps to improve their health.
Secure messaging between providers and patients, and patients’ access to their own medical record (systematic review) [7]	Data exists to support a positive support between the use of a patient portal and the improvement of glucose outcomes and patient satisfaction.
Electronic patient portals: evidence on health outcomes, satisfaction, efficiency, and attitudes: a systematic review [11]	The systematic review shows that patient portals improve patient health outcomes. There are concerns regarding the high cost of the patient portal and the low utilization by patients.
Patient-provider communication and trust in relation to use of an online patient portal among diabetes: the diabetes and aging study [21]	Patients who trusted their health care providers were more likely to use the secure messaging application of the patient portal.
Patient-generated secure messages and eVisits on a patient portal: are patients at risk [27]?	Secure messages and eVisits are intended for low-risk symptoms and regular queries. Over 75% of the patients used these services for the intended purpose, but some used these services to communicate high-risk symptoms, such as chest pain. Services should be expanded and monitored 24/7 in order to expedite the response time.
Parents’ perceptions of a patient portal for managing their child’s chronic illness [25]	Portals seemed to remove barriers to communication, reduced hassle, maximized convenience, and provided a sense of control and independence, reducing anxiety, and providing reassurance.
Consumers’ perceptions of patient-accessible electronic medical record [35]	Low-education, English-speaking health care consumers (n=28) were queried in four focus groups in New York City on perceptions of utility and value of patient portals. Most demonstrated high levels of enthusiasm about the portal’s utility and value. Researchers noted that designers of portals must consider low reading levels and ease of use in order to capture enthusiasm and move the portal movement forward.
Understanding patient portal use: implications for medication management [36]	Portal users demonstrated better A1C (blood sugar) (*P*=.02). Users reported frequent use of medication refill capability, and they were enthusiastic about refill reminders. Portal users were more likely to be Caucasian/white (*P*<.001), have higher incomes (*P*=.005), be privately insured (*P*<.001), and have more education (*P*=.05). Patients uses the portal to manage medication refills and adherence. Additional focus on education may be necessary to reach non-white, low income, and underinsured.
Patient experiences with full electronic access to health records and clinical notes through the My Health*e*Vet Personal Health Record Pilot: qualitative study [37]	Patients reported positive experiences with the transparency that the portal provided. Viewing their records seemed to improve patient empowerment and engagement in their own medical decisions.
Does the use of consumer health information technology improve outcomes in the patient self-management of diabetes? A meta-analysis and narrative review of randomized controlled trials [6]	Health information technology improves patient self-management of diabetes. Further research needed to study the effectiveness of the technology.
Impact of patient use of an online patient portal on diabetes outcomes [14]	Results indicated that patients who access a patient portal were more likely to achieve their target A1C.
Mobile and ubiquitous architecture for the medical control of chronic diseases through the use of intelligent devices: Using the architecture for patients with diabetes [15]	Using the mobile monitoring apps allow patients to access their patient portal at their own convenience. Patients enjoyed the ease of use and the real-time functionality of the portal.
Family perceptions of the usability and value of chronic disease web-based patient portals [17]	Parents agreed that data displayed by the portal was accurate, timely, and useful. Confidentiality was not a major concern. The portal augmented understanding of their child’s condition and their ability to manage it.
Family perceptions of the usability and value of chronic-disease, web-based patient portals [23]	Parents of patients perceived the portal as useful, accurate and timely. Parents using the portal felt confident in the confidentiality of their child's information on the portal.
Technology-assisted patient access to clinical information: an evaluation framework for Blue Button [38]	The implementation of Veterans Affairs (VA) Blue Button is a landmark event for both patients and the VA as an organization. Designers should focus on ease-of-use, low medical literacy, and carefully evaluate potential unintended consequences.
Evaluating user experiences of the secure messaging tool on the Veterans Affairs’ patient portal system [39]	Patients reported positive experiences with increased communication through the VA’s My Health*e*Vet portal. In order to capitalize on this positive enthusiasm, designers should focus on marketing, education, skill-building, and associated system modifications.
The effects on health behavior and health outcomes of Internet-based asynchronous communication between health providers and patients with a chronic condition: a systematic review [40]	Any effect of asynchronous communication enabled through a portal is not clearly demonstrated among the chronically ill sample of patients in this study. Patients seemed to appreciate the secure messaging capability, and they are willing to take initiative to discuss health issues with their providers. Results were not significant.
Impact of patient access to Internet health records on glaucoma medication: randomized controlled trial [41]	Patients with access to an Internet-based glaucoma care support system on glaucoma use demonstrated significant improvement (*P*=.0002) in appropriate use of glaucoma medication, resulting in lower intraocular pressure. While this finding is not directly a patient portal, it does demonstrate a higher level of patient involvement and better outcomes with access to clinical data and care support through the Internet.

^a^Additional articles, beyond the 27 referenced in the text, were added in the peer-review process.

**Table 2:** Affinity matrix illustrating the frequency of factors identified in the literature (n=27).

Factor		Occurrences	Instances of the barrier n (%)
**+ (positive)**			
	Patient-provider communication	[4],[5],[7],[15],[16],[18],[19],[23],[24],[25],[26]	11 (41%)
	Secure messaging	[4],[5],[7],[12],[18-20],[23],[24],[25]	10 (37%)
	Quality of care	[4],[5],[7],[13],[16],[18],[20],[27],[23],[25]	10 (37%)
	Disease outcomes	[4],[5],[6],[7],[12],[15],[16],[21],[30],[23]	10 (37%)
	Educational resources	[4],[7],[12],[13],[16],[18],[21],[28],[23]	9 (33%)
	User-friendliness	[4],[13],[15],[21],[28],[23],[26]	7 (26%)
	Time	[5],[7],[15],[20],[25]	5 (19%)
**− (negative)**			
	Security	[7],[8],[10],[12],[15],[16],[20],[27],[29],[24],[25]	11 (41%)
	User-friendliness	[6],[8],[9],[10],[16],[17],[18],[21],[30],[28],[29]	11 (41%)
	Secure messaging	[8],[10],[20],[27],[29]	5 (19%)
	Time management	[7],[9],[27],[30],[23]	5 (19%)
	Cost	[7],[8],[14]	3 (11%)
	Patient-provider communication	[10],[19],[27]	3 (11%)
	Educational resources	[9],[10],[15]	3 (11%)
	Disease outcomes	[4],[5]	2 (7%)

## Discussion

### Principal Findings

In this systematic review, the authors sought to understand the characteristics of patient portals that cause mixed feelings among patients and providers using patient portals. The authors identified the shared characteristics of patient portals that received favorable responses from patients and providers. The authors also identified the elements that patients and providers believe need to be improved or included in the portal design.

Successful patient portals and Web-based portals are user-friendly and empower patients to take responsibility for managing their health. However, it is evident from the literature reviewed in this study that attitudes toward patient portals differ. There is a lack of clarity regarding the portal design used among respondents; it is unknown whether the portals are designed the same, or whether they differ from one to another. For instance, while one portal may offer immediate access to laboratory results, it may not provide the patient with explanatory material to educate the patient on the meaning of his/her lab results. Another portal may provide an explanation of the lab results but the medical terminology used may cause further confusion for the patient.

Although this manuscript examines the same overall topic of portals from Ammenwerth et al (2007), our findings differ in many ways [31]. Ammenwerth et al did not find statistically significant effect of the portal on medical outcomes. Our study identified positive disease outcomes across 10 of 27 articles (37%) [4-7,12,15,16,21,23,30]. Ammenwerth et al reviewed only randomized controlled trials, however, and they did not broaden their search to encompass the breadth of this study. A common thread through the Ammenwerth et al study and ours is that security concerns rank very highly in the list of negative perceptions about patient portals. This manuscript did not set out to duplicate the work of Ammenwerth, but their work is raised for comparison purposes.

Patients are concerned about the safety of secure messaging on an Internet application, the complexity of the portal design, the lack of guidance in how to use the portal, and the inability to understand the information presented in the educational resources. Patients over the age of 65 years are more likely to have trouble using advanced technology than patients who are more technologically inclined. The gap among users who have different levels of expertise in using advanced technology is called the “digital divide”. The authors believe that by providing patients with a tutorial prior to using the patient portal, patients who have little knowledge of technology will better understand how to operate the portal.

A recurring theme in the literature is the inability of patients to understand medical terminology presented in the patient portal and not being knowledgeable about their own condition. Some patient portals offered a Health Library, which is an interactive educational resource enabling patients to have a bettering understanding of their conditions and how to better manage their health. The resources educate patients on the importance of taking their prescribed medications and changing their behavior in order to improve their health. An advantage of using electronic educational resources is that by providing an electronic version of an information pamphlet covering a patient’s condition, patients will no longer be at risk of losing their information packet.

Patient portals are an effective way to improve communication between patients and their health care providers. However, the large volume of electronic messages sent to providers from their patients may overwhelm providers who must respond to the messages as well as conduct office visits during their workday. In addition to being very expensive to install and maintain, patient portal systems require training for providers who may or may not be willing to shift from paper records to electronic health records. The ease of learning a new technology for the provider is, no doubt, an important factor in the acceptance and adoption of patient portals in health care organizations.

### Conclusions

Innovations in health information technologies improve the quality of and access to health care. Web-based portals provide patients with access to their health record, improve the patient-provider communication, and enable patients to take control of their chronic condition(s). In order to enable the acceptance of patient portals among health care organizations, the portals must be redesigned to be both user-friendly and aesthetically appealing. The authors suggest that a standard patient portal design providing patients with the resources to understand and manage their chronic condition(s) will promote the diffusion of this important technology.

## Abbreviations

EHR: electronic health record
HIT: health information technology
HITECH: Health Information Technology for Economic and Clinical Health
PHR: personal health record
